# Fear, foraging and olfaction: how mesopredators avoid costly interactions with apex predators

**DOI:** 10.1007/s00442-018-4133-3

**Published:** 2018-04-13

**Authors:** Peter M. Haswell, Katherine A. Jones, Josip Kusak, Matt W. Hayward

**Affiliations:** 1 School of Biological Sciences, Bangor University, Bangor, Gwynedd LL57 2UW UK; 2 UK Wolf Conservation Trust, Butlers Farm, Beenham, Berkshire RG7 5NT UK; 3 Department of Biology, Veterinary Faculty, University of Zagreb, Heinzelova 55, 10000 Zagreb, Croatia; 4 School of Environment Natural Resources and Geography, Bangor University, Bangor, Gwynedd LL57 2UW UK; 5 Centre for African Conservation Ecology, Nelson Mandela Metropolitan University, Port Elizabeth, South Africa; 6 Centre for Wildlife Management, University of Pretoria, Pretoria, South Africa

**Keywords:** Mesopredator release, Risk, Giving-up density, Gray wolf, Red fox

## Abstract

Where direct killing is rare and niche overlap low, sympatric carnivores may appear to coexist without conflict. Interference interactions, harassment and injury from larger carnivores may still pose a risk to smaller mesopredators. Foraging theory suggests that animals should adjust their behaviour accordingly to optimise foraging efficiency and overall fitness, trading off harvest rate with costs to fitness. The foraging behaviour of red foxes, *Vulpes vulpes*, was studied with automated cameras and a repeated measures giving-up density (GUD) experiment where olfactory risk cues were manipulated. In Plitvice Lakes National Park, Croatia, red foxes increased GUDs by 34% and quitting harvest rates by 29% in response to wolf urine. In addition to leaving more food behind, foxes also responded to wolf urine by spending less time visiting food patches each day and altering their behaviour in order to compensate for the increased risk when foraging from patches. Thus, red foxes utilised olfaction to assess risk and experienced foraging costs due to the presence of a cue from gray wolves, *Canis lupus*. This study identifies behavioural mechanisms which may enable competing predators to coexist, and highlights the potential for additional ecosystem service pathways arising from the behaviour of large carnivores. Given the vulnerability of large carnivores to anthropogenic disturbance, a growing human population and intensifying resource consumption, it becomes increasingly important to understand ecological processes so that land can be managed appropriately.

**Electronic supplementary material:**

The online version of this article (10.1007/s00442-018-4133-3) contains supplementary material, which is available to authorized users.

## Introduction

Direct interactions between predators and other species can lead to indirect consequences further down the food web via trophic cascades (Ripple et al. 2016). Direct predation as well as behavioural/trait-mediated mechanisms can be important drivers of such processes (Beckerman et al. 1997; Schmitz et al. 2004; Trussell et al. 2006). Evidence for trophic cascades stemming from large carnivores is growing (Ripple et al. 2014); however influence strength and study validity are hotly debated (Allen et al. 2017; Kauffman et al. 2010; Newsome et al. 2015). Understanding the importance of trophic interactions is a fundamental ecological question (Sutherland et al. 2013). Understanding mechanisms, consequences and behavioural responses to predation pressure are crucial first steps in understanding the importance of trophic interactions.

Mesopredator release describes the increase of mesopredator populations after a decline in larger, apex predators (Crooks and Soulé 1999; Soulé et al. 1988). Intraguild predation, competitive killing and interference competition are common where niches overlap (Lourenco et al. 2014; Palomares and Caro 1999; Ritchie and Johnson 2009). Interference interactions from larger carnivores pose a risk to smaller mesopredators and may ultimately affect population demography (Linnell and Strand 2000). Apex predators do not always suppress spatial occupancy and mesopredator abundance (Lesmeister et al. 2015; Lyly et al. 2015). However, continent-wide patterns of mesopredator release have been identified (Letnic et al. 2011; Newsome and Ripple 2014; Pasanen-Mortensen and Elmhagen 2015). Suppressive interactions between carnivores combined with bottom-up effects of environmental productivity can ultimately drive predator and prey species abundance (Elmhagen et al. 2010; Elmhagen and Rushton 2007).

Gray wolves, *Canis lupus* have been observed to kill and chase foxes (Mech and Boitani 2005, p. 269). Some evidence also suggests wolves may contribute to the control of red fox, *Vulpes vulpes* populations (Elmhagen and Rushton 2007). In much of eastern and southern Europe, red foxes co-occur with wolves (Hoffmann and Sillero-Zubiri 2016; Mech and Boitani 2010). A negligible presence of fox hair in wolf diet suggests foxes are not regularly eaten by wolves in Europe (Krofel and Kos 2010; Stahlberg et al. 2017; Štrbenac et al. 2005). Low mortality could reflect effective avoidance of larger predators (Durant 2000). However, interspecific killing may of course occur without consumption (Murdoch et al. 2010). Even in the absence of direct killing, it is plausible that wolves may still behaviourally suppress red foxes through harassment, injury and fear of encounters. Literature suggests minimal dietary overlap between the two carnivores (Bassi et al. 2012; Patalano and Lovari 1993). Competition for landscape features such as den sites, scavenging opportunities and kleptoparasitism however, could still yield negative interactions. Conversely, foxes scavenge from wolf kills in Europe (Selva et al. 2005; Wikenros et al. 2014), suggesting they may exhibit positive behavioural responses toward the species presence even where kleptoparasitism might be risky.

Foxes alter their behaviour in response to the presence of larger carnivores, habitat features and hazardous objects (Berger-Tal et al. 2009; Hall et al. 2013; Vanak et al. 2009). This suggests they are capable of assessing and responding to environmental risk cues. Red foxes have well-developed sensory systems and are known for their flexible behaviour, diet and ability to thrive in anthropogenic landscapes (Bateman and Fleming 2012; Lesmeister et al. 2015; Randa et al. 2009). Olfaction plays an important role in detecting scavengeable food sources (Ruzicka and Conover 2012) and logic suggests it would also play an important role in risk evaluation. A wealth of research exists supporting the recognition and behavioural response of prey species towards odours of their predators (Apfelbach et al. 2005). However we know of only two studies examining the influence of olfactory predation risk cue’s on food harvest by red foxes under the giving-up density (GUD) framework (Leo et al. 2015; Mukherjee et al. 2009). We expanded upon this knowledge by investigating the role of urine in risk analysis and studying behavioural responses in order to explain changes in food harvest.

When responding to predation risk, foragers must trade-off the fitness benefits of avoiding predators with the costs of avoidance in any given context (Brown and Kotler 2007; Brown et al. 1999; Haswell et al. 2017). The better an individual animal is at assessing risk, the more effectively it can forage, balance its energetic cost-benefits and the greater its overall fitness. Methodologies developed by Brown (1988; 1992) and Mukherjee et al. (2009) were adapted to investigate fox giving-up densities (GUDs) and foraging behaviour (methodological considerations, online resource 1). A GUD is the amount of food left behind in a given food patch after the forager quits the patch (Brown 1988). As a forager devotes time to harvesting a food patch (assuming it is depletable), the available resources decline as does the harvest rate (Brown 1988). Foragers should leave a given patch once the harvest rate (*H*) is equal to the sum of the metabolic costs (*C*), predation costs (*P*) and missed opportunity costs (MOC) i.e. *H* = *C* + *P* + MOC (Brown 1988; Shrader et al. 2012). By holding other parameters constant between food patches, it is possible to investigate species or habitat specific differences in predation cost (Brown 1988). Increases in predation risk should increase the GUD with animals foraging less in risky patches (Brown 1988). GUDs can help measure the response of organisms to olfactory cues and their perception of the predation costs (*P*) associated with foraging, thus illuminating ecological processes.

Understanding the contribution of different biodiversity components to ecosystem functioning is vital (Sutherland et al. 2013). Suitable scientific information becomes especially essential if wildlife is to be properly managed in public trust (Treves et al. 2017). The existence of mesopredator release has become more widely supported (Newsome et al. 2017; Ritchie and Johnson 2009), yet understanding of the mechanisms and processes are still needed if the consequences of anthropogenic intervention are to be fully understood. Furthermore, cross-context assumptions should be avoided and there is still great need to understand the impacts of large carnivores for any given system (Haswell et al. 2017; Kuijper et al. 2016). This paper examined red fox foraging behaviour in response to an olfactory risk cue (wolf urine) in order to test the importance of olfaction in risk analysis, identify any resultant suppression and the foraging strategies employed where apex predators pose risk.

## Methods

### Study site

Plitvice Lakes National Park (PLNP) is in the Dinaric Alps, Croatia between 44°44′34″ and 44°57′48″N and 15°27′32″ and 15°42′23″E (Šikić 2007). The park (297 km^2^) is a mosaic of mountains and valleys with altitude ranging from 367 to 1279 m above sea level (Romanic et al. 2016). The karst (limestone and dolomite) landscape of the park is characterised by underground drainage systems, sink holes and caves, and contains ~ 1% surface water with a series of streams, rivers, lakes and waterfalls (Šikić 2007). Topography can influence microclimates within the park but in general, summers tend to be mild and sunny and winters long with heavy snowfall; temperatures range between winter lows of − 3 °C and summer maximums of 36 °C and annual precipitation is 1550 mm (Šikić 2007).

Romanic et al. (2016) estimate approximately 1770 people occupy 19 settlements within the park’s boundaries. Being a national park, the only economic uses permitted within the boundaries are tourism and recreation (Firšt et al. 2005).

Between July and September 2015, foraging experiments were conducted within the mixed beech (*Fagus sylvatica*) and fir (*Abies alba*) forests of PLNP. Forest roads were surveyed for carnivore signs with the assistance of a detection dog ≥ 1 week prior to the experiments—maximising data yield by selecting sites with fox presence. During surveys the dog did not leave the road. Population density of red fox in Croatia is estimated at 0.7 animals per km^2^, with a territory size of 1.43 km^2^ per fox (Galov et al. 2014; Slavica et al. 2010). Home ranges between fox group members can often overlap (30–100%) (Poulle et al. 1994). Fox individuals could not be identified by pelage markings but distance between sites (≥ 1.5 km) ensured site independence and was greater than distances previously used (e.g., Leo et al. 2015; Mukherjee et al. 2009). Twelve sites were attempted. In early July, foxes foraged from three of those sites in the north-west of the park; a less accessible area, partly open to hiking and local traffic but receiving far fewer tourists than the lakes. These sites were then repeated in late August to give a better temporal representation of response consistency.

### GUD methodology

Feeding stations were positioned similarly to those used by Altendorf et al. (2001) with each site consisting of a 2 × 3 grid with six food patches spaced 60 m apart. Patches were placed in woodlands, with three patches on either side of an unpaved forest road to maximise detection likelihood and keep road related risk consistent. Each food patch contained twenty 4 g dog food pieces (80 g per patch, Bakers Complete Meaty Meals Chicken), systematically mixed in 8 L of local substrate put through a 5 mm sieve and placed inside a 14 L bucket half submerged in the ground. To increase detection of the food patches by foragers, 5 ml of liquid leached from raw meat was applied to the surface of the soil within the bucket each day. We measured GUDs and replenished food pieces daily. Sites were visited in the hottest parts of the day (afternoon) to ensure foragers were not disturbed.

To standardise harvest rate (*H*), the structure of artificial patches was kept consistent (substrate and food). The substrate to food ratio was chosen after trials with less soil were harvested completely and trials with more soil were harvested minimally (PMH unpubl. data). A decline in harvest rate over time was thus ensured through the use of a depletable food source in a suitable volume of inedible soil matrix (Bedoya-Perez et al. 2013; Brown 1988). Six food patches were available to the same forager to ensure consistent missed opportunity costs (MOC). Patch consistency kept energetic costs (*C*) consistent and data collection occurred during typical summer weather conditions. Habitat-associated risks were kept somewhat consistent by using just mixed beech and fir woodlands. Although not explicitly mentioned in earlier studies (Leo et al. 2015; Mukherjee et al. 2009), the influence of human scent contamination was minimised during data collection by wearing thick gloves, a mouth mask and long sleeved clothes kept in the presence of the liquid leached from meat rather than smelling of detergent. Predation costs (*P*) were manipulated using scent treatments.

Foxes foraged from feeding stations within a day during pilot studies (PMH unpubl. data). The first day of the 11-day experimental cycle was untreated to provide an opportunity for detection and acclimatisation. A control scent consisting of 25 g of sand scented with 3 ml of mint extract (Asda extra special peppermint extract) was spread across a piece of locally sourced moss (15 × 15 cm) placed on the ground 15 cm to the north of the bucket on day 2 and left during the remaining control-treatment days. On day 7, the control treatment was removed from all patches and 25 g of granules scented with wolf urine (PredatorPee^®^, Wolf Urine Yard Cover Granules) were placed on fresh moss in the same location as the procedural control. Throughout the 5-day treatment periods, both odours and volumes used were detectable by researchers.

Daily replenishment of GUDs should result in higher predictability and exploitation of patches by foragers in what has been termed the “magic pudding” effect (Bedoya-Perez et al. 2013). An 11-day window was used for each experiment to reduce the likelihood of foragers becoming over-reliant upon predictable food patches. We deemed that there was less expectation of a response to wolf urine given its application later in the test procedure when foxes would be more familiar and reliant upon food patches. Thus, the experimental approach was considered conservative.

During the experiment, automated cameras were set to record 30-s videos with 30-s intervals. Cameras were positioned 0.4 m high on trees 2 m from feed stations and angled to ensure buckets were in central view. Camera-traps permitted accurate species identification of those responsible for the GUDs as well as the collection of additional behavioural data.

### Additional variables

Soil penetration could affect GUDs if some substrates were harder to dig through than others. This was measured by dropping a wooden 1 m ruler into the bucket from shoulder height and measuring the depth that the ruler penetrated the soil.

A photograph was taken from each GUD patch towards the road, 30 m away. Photos were taken consistently with a 3 megapixel camera always fully zoomed out. A systematic grid sample of 100 pixels (10 × 10) was analysed from each photograph (0.003% of pixels). Pixels were assigned to categories of open (no material blocking view to the road) or other (biotic or abiotic material) to calculate the percentage visibility to the road (number of open pixels) at each location. Pictures were analysed using SamplePoint V1.58—a method that provides accuracy comparable with field methods for ground cover measurements (Booth et al. 2006).

Data for the fraction of moonlight illuminated at midnight were obtained from the US naval observatory (http://aa.usno.navy.mil/data/index.php). Due to each experiment day beginning one afternoon and running overnight until the next afternoon, an experimental day beginning on the afternoon of June 26th and finishing on the afternoon of June 27th for example, was ascribed “moonlight data” from midnight on June 27th.

### GUD analysis

Camera-trap videos were used to identify the last known forager and assign GUD data for each experiment day. On rare occasions where cameras failed to trigger but the patch had been visited (*N* = 8 from 195 total GUDs), field signs were used to confirm fox visits. GUD scores were assigned to foxes when they were the last species identified foraging at the patch (every occasion foxes visited) with the exception that once a patch was discovered by foxes, all following days where a visit was not recorded were assigned the maximum GUD of 20 to ensure data reflecting patch avoidance was also included. Foxes were captured on video during both scent treatments for all sites, so death of subjects could be ruled out.

Following Leo et al. (2015), we treated GUDs as count data. The counts were commonly occurring (food pieces were often left behind resulting in higher GUDs) and, as such, a negative binomial regression (negative binomial distribution with a log link) generalized linear mixed model (GLMM) was used to examine the influence of independent variables upon GUDs (Heck et al. 2012). All analysis was conducted in IBM SPSS Statistics 22. The fixed effect was scent treatment. Covariates were percentage visibility to the road, soil penetration (cm) and fraction of the moon illuminated. The repeated measures aspect of data points from the same patch and a random effect for patch location were also included. Robust standard error estimation was used to handle any violations of model assumptions and the Satterthwaite approximation was applied to denominator degrees of freedom (few level 2 units, unbalanced data and more complex covariance matrices).

### Behavioural analysis

The number of visits and total visit duration per experiment day was extracted from the videos. New visits were considered to begin if the period between two videos was greater than 15 min. Visit duration was recorded as the amount of time in seconds from the beginning of the first video and the exact time the fox (any body part) was no longer visible on the last video for that visit. The influence of scent treatment, percentage visibility to the road, soil penetration and fraction of the moon illuminated upon total visit duration was analysed with a negative binomial regression GLMM. Visit frequency per experiment day was analysed with a loglinear (Poisson distribution and log link) GLMM. All other model parameters were the same as for the GUD analysis.

Where foxes visited patches, behavioural data were extracted from videos taken by automated cameras using Solomon Coder Beta 15.11.19. Strict definitions of behaviours were described in an ethogram (online resource 2). Given that identification of most behaviour required the orientation of the head or neck to be identifiable, the length of videos was recorded as only the duration during which the animals head orientation was identifiable i.e. once the head and neck had left the visible field, video timing stopped. Videos where animals were not present throughout the entirety of the 30-s video did not then skew the data. Duration of time spent engaging in major and minor vigilance, foraging from the bucket and sniffing the ground were extracted from each video. Percentage of time spent enacting behaviours [(total behaviour duration/total video length) × 100] was calculated for each patch and experiment day. Percentage of time spent enacting behaviours were analysed with negative binomial regression GLMMs. All other model parameters were the same as for the GUD analysis.

### Quitting harvest rate curves

Following the protocol of Kotler et al. (2010) quitting harvest rates (QHR) were calculated for each treatment. Overall handling time (*h*) was estimated with Kotler and Brown’s (1990) multiple regression equation derived from Holling’s (1959) disc equation: *t* = (1/*a*) [ln (*N*_0_/*N*_f_)] + *h* (*N*_0_−* N*_f_). *t *= the total time spent at patches (visit durations obtained from camera trap footage), *a *= attack rate*, N*_0_ = Initial amount of dog food pieces in the patch (20) and *N*_f_ = the GUD. Two variables, ln (*N*_0_*/N*_f_) and (*N*_0_−* N*_f_) were created, these variables were then regressed against values for *t*, the coefficients of which yielded estimates for 1/*a* and *h*, respectively.

We then used *h,* in this case 16.79 s/food piece to create a new variable *t*_*new*_ [*t*_*new*_ = *t *− *h* (*N*_0_−* N*_f_)]. Using the regression *t*_new_ = (1/*a*) [ln (*N*_0_/*N*_f_)], subsets of values for *t*_new_ and [ln (*N*_0_/*N*_f_)] were then used to obtain coefficients giving estimates for 1/*a* and thus *a* (1/coefficient value = *a*) for each scent treatment. Estimates of *h* and treatment specific *a* were then used in Hollings disc equation to calculate QHR for each resource density (1–20 food pieces): QHR = (a*GUD)/(1 + a*h*GUD). Mean GUDs were also used to obtain a characteristic QHR for each treatment. In order to fully characterize risk management strategy, the treatment specific harvest rate curves and QHR for mean GUD’s were then plotted.

## Results

### GUDs

A total of 195 fox GUD measures were obtained. Even with a conservative experimental approach (less expectation of a response to wolf urine given its application later in the test procedure when foxes would be more familiar and reliant upon food patches), there was a significant effect of scent treatment upon GUDs (*F*_1,93_ = 17.243, *P* < 0.001). GUDs were significantly higher (less food harvested from patches) during wolf urine treatment (14.98 ± 6.94 SD, *N* = 127) than under the control treatment (mint, 11.16 ± 7.10 SD, *N* = 68). Soil penetration (*F*_1,45_ = 0.376, *P* = 0.54), percentage visibility to road (*F*_1, 5_ = 2.629, *P* = 0.17) and fraction of the moon illuminated (*F*_1,38_ = 0.747, *P* = 0.39) did not have a significant effect on GUDs.

### Behavioural analysis

#### Visit duration and frequency

**Fig. 1:**
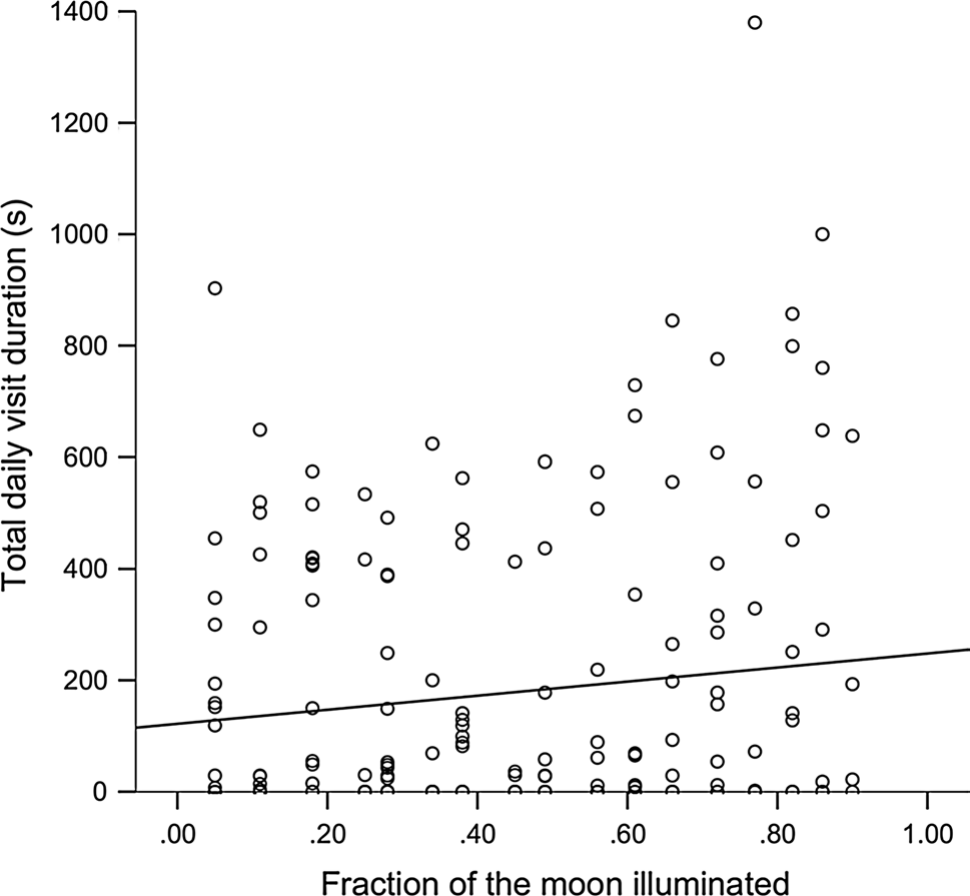
Total visit duration by red foxes, *Vulpes vulpes*, to food patches each day had a positive relationship with fraction of the moon illuminated

In total, 790 videos of fox visits were used to calculate total visit duration (s) for 187 experiment days (camera malfunctions excluded *N* = 8). Scent treatment had a significant effect on total daily visit duration to the feeding patches (*F*_1,9_ = 10.570, *P* = 0.01). Visits were longer under the control scent (mint, 269.14 ± 307.22 SD, *N* = 63) than with wolf urine (132.59 ± 212.47 SD, *N* = 124). Soil penetration (*F*_1, 10_ = 0.279, *P* = 0.61) and percentage visibility to road (*F*_1,6_ = 1.396, *P* = 0.28) did not have a significant effect on total daily visit duration. Even though moonlight levels did not affect GUDs, total daily visit duration had a positive relationship with fraction of the moon illuminated (*F*_1,11_ = 7.388, *P* = 0.021, Fig. 1). No independent variables significantly influenced visit frequency per experiment day.

#### Percentage of time spent enacting behaviours

**Fig. 2:**
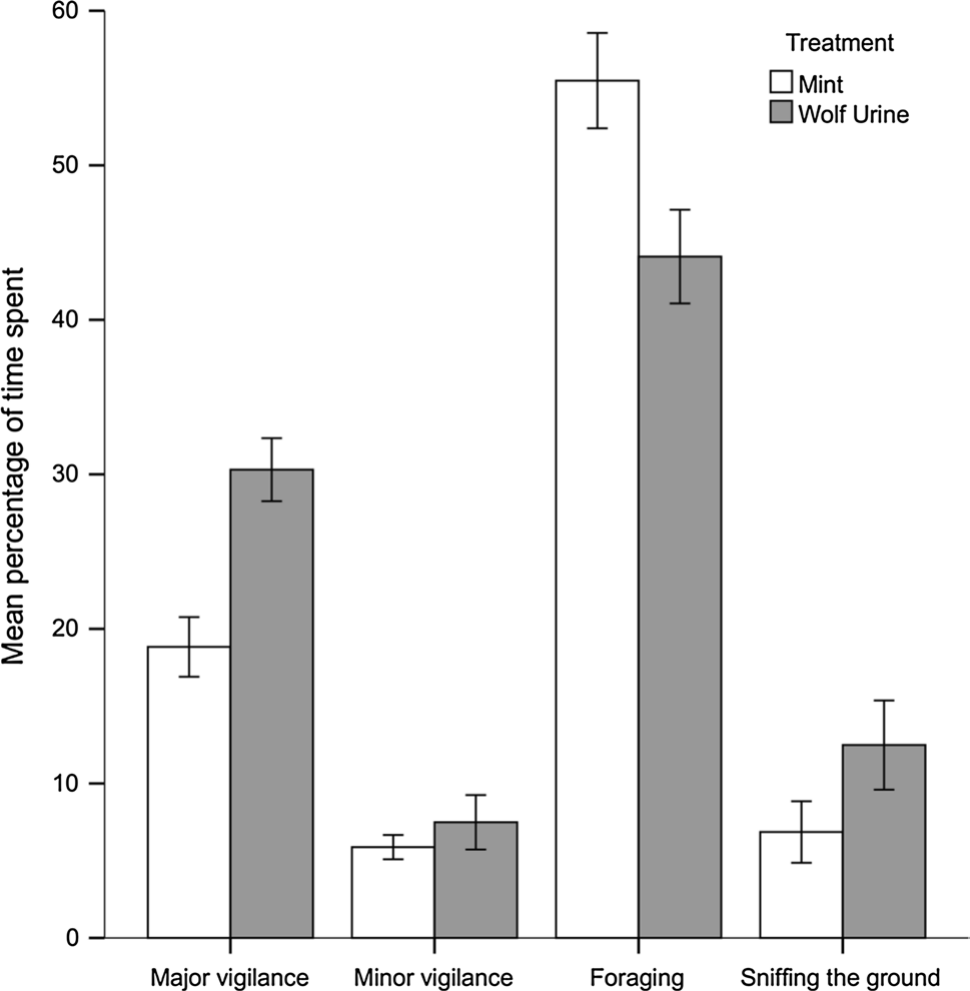
Mean percentage of time spent by red foxes enacting major vigilance (mint, 18.83 ± 13.37 SD, *N* = 48, wolf urine, 30.30 ± 16.56 SD, *N* = 66), minor vigilance (mint, 5.88 ± 5.44 SD, *N* = 48, wolf urine, 7.48 ± 14.33 SD, *N* = 66), foraging (mint, 55.48 ± 21.38 SD, *N* = 48, wolf urine, 44.09 ± 24.64 SD, *N* = 66) and sniffing the ground (mint, 6.85 ± 13.80 SD, *N* = 48, wolf urine, 12.48 ± 23.46 SD, *N* = 66) at artificial feeding stations during two scent treatments, a control (mint) and wolf urine. Error bars represent ± 1 SEM

Behaviour was identifiable from 782 of the 790 videos of fox visits, providing behavioural data for 114 experiment days (72 patch avoidance days with no videos, 8 days with camera malfunctions, and 1 day with fox on video but behaviour identification not possible due to head being out of view). At patches, foxes spent significantly more of their time enacting major vigilance during wolf urine treatment than when the control scent was present (*F*_1,26_ = 31.996, *P* < 0.001, Fig. 2). Soil penetration (*F*_1,9_ = 3.679, *P* = 0.087), percentage visibility to road (*F*_1,8_ = 0.037, *P* = 0.85) and fraction of the moon illuminated (*F*_1,104_ = 2.493, *P* = 0.12) did not have a significant effect. No independent variables had a significant effect upon time spent enacting minor vigilance.

Foxes spent significantly less of their time foraging at patches with wolf urine than with the control (*F*_1,52_ = 6.132, *P* = 0.017, Fig. 2). Soil penetration (*F*_1,24_ = 2.128, *P* = 0.16), percentage visibility to road (*F*_1,6_ = 0.847, *P* = 0.39) and fraction of the moon illuminated (*F*_1,29_ = 0.121, *P* = 0.73) did not have a significant effect.

**Fig. 3:**
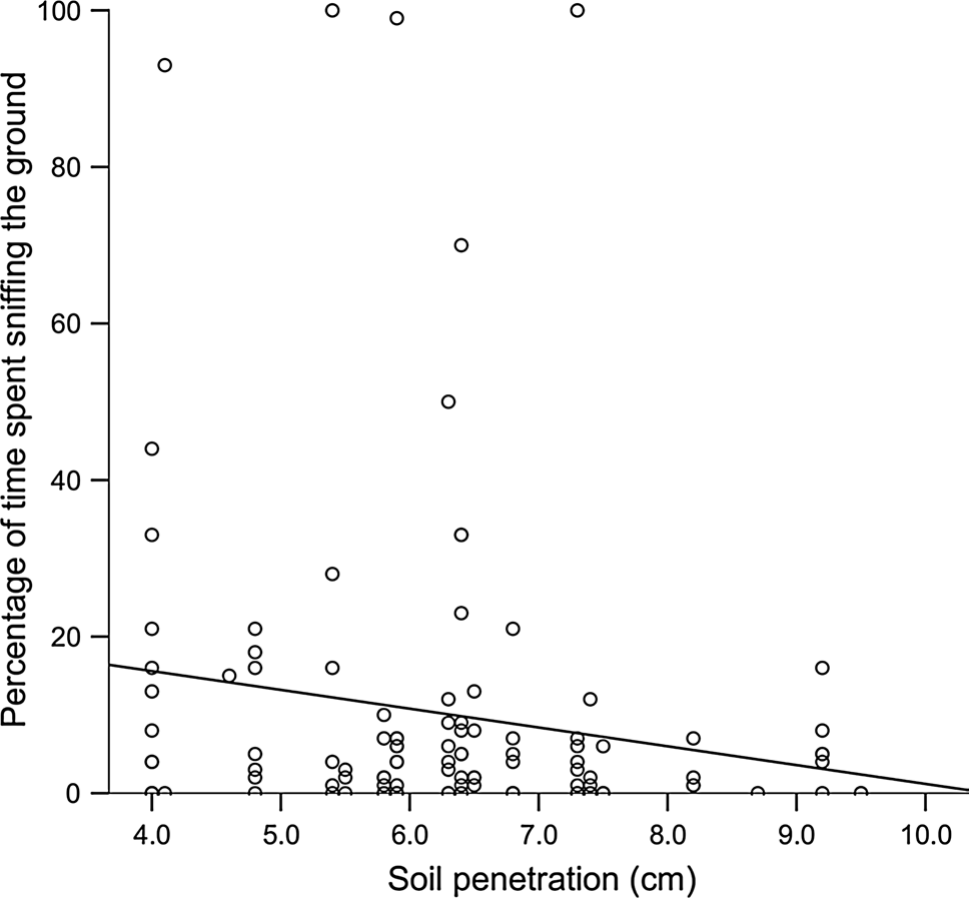
Percentage of time spent by red foxes sniffing the ground had a negative relationship with soil penetration

When at patches, foxes spent significantly more of their time sniffing the ground during wolf urine treatment than the control (*F*_1,44_ = 5.381, *P* = 0.025, Fig. 2). Percentage of time spent sniffing the ground had a negative relationship with increasing soil penetration (*F*_1,4_ = 20.530, *P* = 0.009, Fig. 3). Percentage visibility to road (*F*_1,5_ = 0.489, *P* = 0.52) and fraction of the moon illuminated (*F*_1,109_ = 2.892, *P* = 0.092) did not have a significant effect.

### Quitting harvest rate curves

**Fig. 4:**
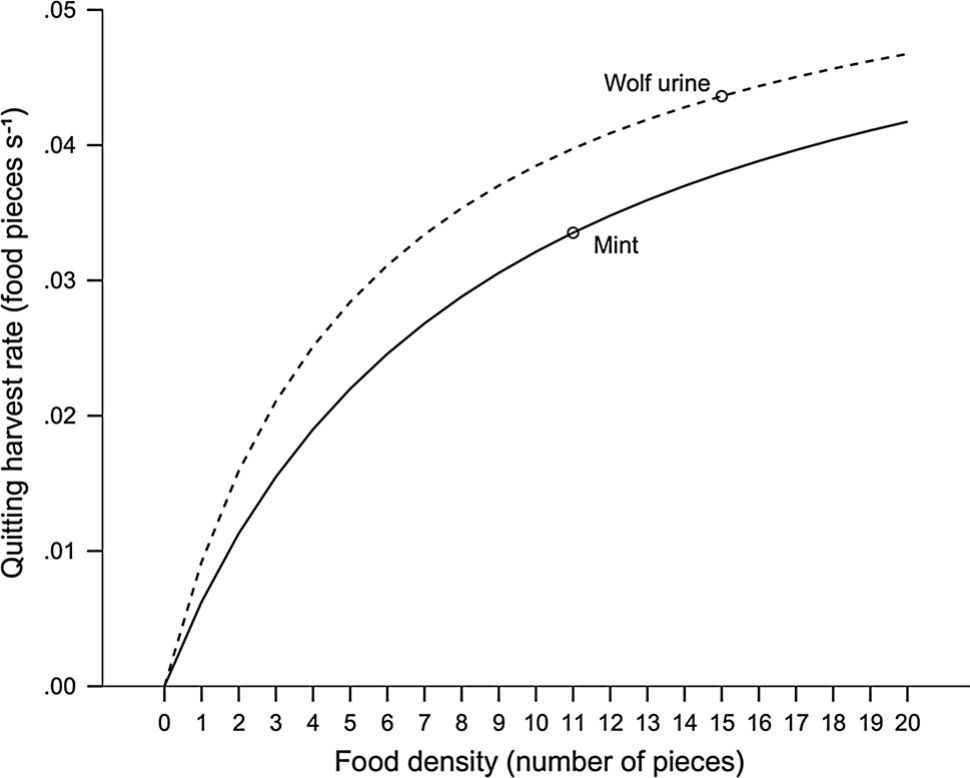
Harvest rate curves for red foxes foraging under two scent treatments, a control (mint, solid line) and wolf urine (dashed line). Quitting harvest rates (QHR) were plotted as a function of the number of food pieces in the patch. Points represent characteristic QHR for mean GUD’s under each scent treatment

Lower mean GUD and characteristic quitting harvest rate (QHR) during mint treatment (0.034 food pieces/s) corresponds with greater time allocation (Fig. 4), as also shown by our analysis of time spent at patches. Higher characteristic QHR under wolf urine (0.044 food pieces/s) suggest foxes required higher remuneration when predation costs were higher. The QHR slope was however steeper and the attack rate higher under wolf urine (10.86 × 10^−3^/s) than under mint treatment (6.97 × 10^−3^/s), indicating quicker food harvest under wolf urine treatment.

## Discussion

We show that wolf urine signifies risk for foxes and olfaction is a mechanism by which foxes assess risk. The behavioural responses of foxes to wolf urine presumably reduced predation risk but also reduced their ability to utilise food resources. These behavioural strategies help explain how foxes are able to persist in sympatry with wolves, but also help explain some of the suppressive impacts wolves have on foxes.

When living in sympatry with larger carnivores, mesopredators often employ strategies such as vigilance, spatial or temporal avoidance, response to risk cues and adjustments in feeding behaviour (Durant 2000; Hayward and Slotow 2009; Wikenros et al. 2014). In the presence of large carnivores, anti-predator strategies permit avoidance of danger but can carry costs such as decreased activity, restricted habitat use and reduced nutrient intake (Hernandez and Laundre 2005; Lesmeister et al. 2015).

At least at a localised scale, wolves negatively affected red fox foraging efficiency with foxes exploiting patches less thoroughly in the presence of wolf urine. Reduction in time spent at patches came at a cost of lower food harvest from patches, with the amount of food left behind (mean GUD) being 34% higher under wolf urine and quitting harvest rates for mean GUDs being 29% larger under wolf urine than under mint treatment. This indicates that foxes required a higher payoff when olfactory cues suggested wolf presence. Such fitness costs of antipredator responses could affect survival and reproduction, ultimately impacting population dynamics (Creel and Christianson 2008). Such processes could contribute to the effect apex predators have on the distribution of mesopredators (Newsome et al. 2017).

Contrary to expectation, additional strategies employed by foxes in response to wolf urine did not come at a cost to harvest rates. Kotler et al. (2010) proposed that a steeper QHR curve (quicker harvest) suggests less time investment in apprehensive behaviours. Our video analysis however shows that foxes spent a significantly greater percentage of time engaging in some forms of apprehension (major vigilance and sniffing the ground) and a lower percentage of time foraging under the wolf urine treatment, yet still achieved higher harvest rates. For some species harvest rates may be a product of more than just time allocation to apprehension and foraging. They may also be affected by how these activities are performed as well as time allocation to different types of apprehensive behaviour and other activities.

Having the head up in major vigilance, permits visual, auditory and scent based detection of danger and likely represents an effective, albeit costly, investment of time spent in risky food patches. Higher levels of predator detection behaviour do not always come at a cost to foraging performance and harvest rates can increase alongside proportion of time spent vigilant (Cresswell et al. 2003). It is feasible that foxes increased their digging speed and encounter rates when foraging under wolf urine in order to compensate for the reduction in time spent foraging.

Foxes were less casual and more focused about how time was spent under wolf urine, investing highly in major vigilance and spending less time engaging in “other” behaviours that were not productive to obtaining food or ensuring safety e.g. masticating without being vigilant (PMH unpubl. data). Mastication could not be measured in a comparable way to the behaviours recorded in this study as the jaws could not always be seen, however we note that, where observable, mastication without vigilance appeared to be the dominant “other” behaviour. Herbivores have been observed to temporally and spatially partition their ruminating behaviour from their foraging behaviour (Lynch et al. 2013; Nellemann 1998). Mesopredators like foxes may also adjust their digestive behaviour in response to predation risk. Foxes may have chewed more quickly, chewed less or even swallowed pieces whole under wolf urine treatment, digesting away from risky patches instead of investing time aiding the digestive process by masticating while at patches. Mastication may also be reduced in risky locations because it can inhibit auditory vigilance (Lynch et al. 2013, 2015).

Mesopredators likely have a more complex olfactory landscape than organisms on the periphery of food webs and behavioural response to scent could be affected by scent strength, integrity and context (Jones et al. 2016). Previous works investigating the response of foxes to alternative risk cues have yielded varying results. Observations of red (Scheinin et al. 2006) and Indian foxes, *Vulpes bengalensis* (Vanak et al. 2009) only showed significant reductions in food bait take in response to direct predator presence (golden jackal, *Canis aureus* and domestic dog *Canis lupus familiaris*, respectively), but not to olfactory risk cues (urine, or scat and urine, respectively).Observations were short and scents fresh so it could be concluded that foxes did not respond to these particular risk cues and only responded to immediate threats, or that foxes in these studies were bigger risk takers than in our study. However, these studies did not follow a GUD framework so responses to scent may have reflected experimental setup more than fox behaviour. Foraging may have been too easy or profitable and food to substrate ratios in these experiments may have only permitted observation of strong responses. Nonetheless, food take and behavioural responses towards live animals in both studies still suggest fearful responses of foxes towards larger predators. The studies also suggest that fearful responses to the actual presence of predators are likely to be stronger than to risk cues alone.

Under a GUD framework, Mukherjee et al. (2009) observed that foxes foraged more from patches with wolf scat present. They suggested that scat may provide information of a predator’s whereabouts and could indicate that a predator has moved on and that the patch in fact carries less risk. The responses observed in this study suggest urine presents a more immediate predator presence cue. Scat can act as a territorial marker and conveyer of information about the depositor (Barja 2009). Peters and Mech (1975) however concluded that raised leg urination was probably the most effective method of territory maintenance. Competitors may associate higher risk with urine than with scat. Canids also preferentially faecal mark on visually conspicuous features, suggesting scat placement is an important aspect of communication (Barja 2009; de Miguel et al. 2009; Hayward and Hayward 2010). Dependent on the context and placement, scat may communicate risk but could also be positively associated with scavengeable food sources.

Mukherjee et al. (2009) also suggested that the lower presence of wolves in the study area and higher presence of the larger striped hyena, *Hyaena hyaena*, could have been responsible for their observations. Aversion to foreign odours likely requires a social unit to have experience of antagonistic events (Peters and Mech 1975). At 1.4–1.6 wolves per 100 km^2^ (JK unpubl. data, estimates based on 100% MCP polygons and snow tracking of two packs utilising PLNP during 2015), wolf density was higher in PLNP than the Croatian average of 1.3 (Štrbenac et al. 2005). Given fox responses to wolf urine and wolf density, encounter rates might also have been higher in PLNP.

Leo et al. (2015) examined fox GUDs in response to a combination of canid body odour (an indicator of close proximity and hence immediate threat) and scat (territorial demarcation and a less proximate threat). GUDs were higher under dingo odour than control treatments. This is unsurprising given the threat dingoes (*Canis lupus dingo*) pose to foxes through direct killing (Marsack and Campbell 1990; Moseby et al. 2009). The dingo has a different ecology to the wolf and exists in unique ecosystems (Mech and Boitani 2005; Purcell 2010). While interactions may vary depending on context, the findings of Leo et al. (2015) suggest that the combination of body odour and scat at locations such as den sites are likely to affect foxes as well.

Context can be an important driver of interspecific relationships between predators (Haswell et al. 2017). The studies discussed suggest that cue type, species composition, experience and demography might be important factors in driving response to risk cues. A forager’s response to risk may also vary dependent on factors such as social structure, food patch quality and energetic state (Fortin et al. 2009; Harvey and Fortin 2013; Hayward et al. 2015). Nonetheless, cues informing of more immediate risk (direct predator presence, urine or body odour) should in general yield stronger behavioural responses. Inferences and responses to olfactory cues will depend upon selection pressures (Jones et al. 2016). Apex predator impacts may be weaker farther away from core areas such as den sites (Miller et al. 2012). The recently proposed “enemy constraint hypothesis” also predicts weaker mesopredator suppression at peripheries of large carnivore range (Newsome et al. 2017). At range edges, reduction in apex predator presence and risk cues would be expected. A reduction in behavioural suppression through mesopredator response to olfactory risk cues would thus also be expected. Factors affecting scent demarcation and landscape use by apex predators should in-turn affect risk perception and behavioural responses of mesopredators.

Suppression by larger predators can affect the abundance and behaviour of mesopredators, often but not always having consequent impacts upon mesopredator prey species (Ritchie and Johnson 2009). Mesopredator response to risk landscapes can have behavioural knock-on effects, influencing landscape and resource use by prey species (Palacios et al. 2016). Predator odours including those of foxes have a range of behavioural and physiological effects upon prey species (Apfelbach et al. 2005). Foxes can also have stabilising effects upon their prey populations (O’Mahony et al. 1999) or interact competitively with smaller carnivores (Bischof et al. 2014; Petrov et al. 2016). Behavioural interactions clearly play a part in maintaining functioning stable ecosystems. Anthropogenic disturbance or direct loss of processes through trophic simplification can however interfere with these complicated systems, leading to problems (Estes et al. 2011; Frid and Dill 2002; Prugh et al. 2009). Removal or disturbance of large carnivores may interfere with behavioural processes which also require consideration when managing human landscape use.

### Data availability

Datasets analysed during the study can be made available from the corresponding author on reasonable request.

## Electronic supplementary material

Below is the link to the electronic supplementary material.

Supplementary material 1 (PDF 84 kb)

Supplementary material 2 (PDF 69 kb)
