# Supplementary material for: Fear, foraging and olfaction: how mesopredators avoid costly interactions with apex predators
Source: Oecologia. 2018 Apr 13;187(3):573–83. doi: 10.1007/s00442-018-4133-3 (PMC6018578; doi:10.1007/s00442-018-4133-3)
Supplement: Supplementary file 1 — Supplementary material 1 (PDF 84 kb) [file 442_2018_4133_MOESM1_ESM.pdf]

Table 1: Considerations proposed for the application of the giving-up density framework in field research and how the methodologies created in this paper accounted for these considerations.

**Considerations**	**Author(s)**	**This study**
Patches aren't natural.	Brown (1988)	Natural substrate (soil), patches were used by foxes during the 2014 pilot study.
Inappropriate food resources offered.		Food was consumed by foxes during the 2014 pilot study.
Foragers may become satiated.		Sargeant (1978) found captive kept adult daily food consumption ranging from 266 – 541g per day. Wild foxes presumably consume more food and each site contained 480g per day. All food was consumed from a site during the 2014 pilot study; the volume of food unlikely satiates as foxes continued to visit before patches were replenished (PMH *unpubl. data*).
Visits by more than one forager.		Camera identification of last visiting species but individual identification by pelage not possible.
Curvilinearity between harvest rate & energy/ diminishing returns.	Bedoya-Perez et al. (2013)	Depletable food in a suitable volume of inedible matrix (pilot study). Patches were only harvested to empty on 11 occasions from 195 GUDs suggesting diminishing returns were experienced.
Energetic state of forager.		Signs of mange or parasite grooming behaviours were not observed from videos. Data collected on multiple occasions. Single national park. No hunting sites or human food subsidies in study area.
Effects of group foraging.		Almost entirely solitary foraging, 2 foxes were only observed on 3 out of 790 videos and even when observed together only the behaviour of one fox at a time was identifiable, i.e. one fox departed from or was only part in camera shot as the other arrived.
Food quality & substrate properties.		Target species utilised both during the pilot study.
Predictability of patch.		Duration was limited to avoid the magic pudding effect. Conservative use of wolf urine as the second treatment. We deemed that there was less expectation of a response to wolf urine given its application later in the test procedure when foxes would be more familiar and reliant upon food patches.
Behavioural traits.		Analysis of behavioural/ temporal strategies from video observations.
Non-target species.		Camera identification of last visiting species.

References

Bedoya-Perez MA, Carthey AJR, Mella VSA, McArthur C, Banks PB (2013) A practical guide to avoid giving up on giving-up densities. Behav Ecol Sociobiol 67:1541-1553. doi: 10.1007/s00265-013-1609-3

Brown JS (1988) Patch use as an indicator of habitat preference, predation risk, and competition. Behav Ecol Sociobiol 22:37-47. doi: 10.1007/bf00395696

Sargeant AB (1978) Red fox prey demands and implications to prairie duck production. J Wildlife Manage 42:520-527. doi: 10.2307/3800813
