# Supplementary material for: Fear, foraging and olfaction: how mesopredators avoid costly interactions with apex predators
Source: Oecologia. 2018 Apr 13;187(3):573–83. doi: 10.1007/s00442-018-4133-3 (PMC6018578; doi:10.1007/s00442-018-4133-3)
Supplement: Supplementary file 2 — Supplementary material 2 (PDF 69 kb) [file 442_2018_4133_MOESM2_ESM.pdf]

Table 2: Ethogram describing behaviours recorded (Bold font). Other behaviours that might accompany or be performed alongside those recorded are described in plain text.

Category	Behaviour	Description / indicator	May include or be accompanied by
**Environmental assessment**	**Major Vigilance**	**The eyes are directed anywhere but the bucket or ground. The top of the head is above the shoulders. The neck is held above the horizontal plane.**	**Tilting of the head to one side. Focused/pricked ears. Mastication (rarely). Locomotion (rarely).**
	**Minor Vigilance**	**The eyes are directed anywhere but the bucket or ground. The top of the head is level with the shoulders. The neck is horizontal.**	**Tilting of the head to one side. Focused/pricked ears. Mastication. Locomotion.**
	Focused/ pricked ears	The ears/pinnae are upright with the inner surface area of the pinnae focused forwards.	
	**Sniffing the Ground**	**The nose is outside of the bucket and lowered 45° or more below the horizontal plane. The neck is angled below the horizontal plane and the eyes are directed at the floor. Not masticating. Nose is ≤15cm (half bucket width) from the ground.**	**Side to side or up down movements of the head. Locomotion.**
**Foraging**	**Foraging from the bucket**	**The muzzle may be inside the perimeter of the bucket or within a buckets width (30cm) of its edge. The neck is angled below the horizontal plane with the eyes directed towards the bucket. The fox may be digging with front paws close to (within 30cm) or within the bucket. It may alternatively be biting the bucket, moving its head around inside the bucket or removing a food item with its jaws.**	**Mastication (rarely), standing.**
	Mastication	Repeated upwards and downwards movement of the lower jaw.	Up down movements of the head.
